# Misdiagnosis and inappropriate treatment of cutaneous leishmaniasis: a case report

**DOI:** 10.1186/s40794-022-00175-5

**Published:** 2022-08-01

**Authors:** Katarzyna Sikorska, Marta Gesing, Romuald Olszański, Anna Roszko-Wysokińska, Beata Szostakowska, Katarzyna Van Damme-Ostapowicz

**Affiliations:** 1 Division of Tropical Medicine and Epidemiology, Department of Tropical Medicine and Parasitology, Institute of Maritime and Tropical Medicine, Faculty of Health Sciences, Medical University of Gdansk, Gdansk, Poland; 2 Department of Infectious Diseases, Faculty of Medicine, Medical University of Gdansk, Gdansk, Poland; 3 University Center for Maritime and Tropical Medicine, Gdynia, Poland; 4 Division of Tropical Parasitology, Department of Tropical Medicine and Parasitology, Institute of Maritime and Tropical Medicine, Faculty of Health Sciences, Medical University of Gdansk, Gdansk, Poland; 5 Department of Health and Caring Sciences, Faculty of Health and Social Sciences, Western Norway University of Applied Sciences, Førde, Norway

**Keywords:** Cutaneous leishmaniasis, Diagnosis, Travel, Treatment, PCR, Case report

## Abstract

**Background:**

Leishmaniasis is a widespread disease in tropical and subtropical countries, except for Australia and Oceania. In Poland, tourists, migrants and travellers from leishmaniasis-endemic countries may carry *Leishmania*.

**Case presentation:**

We present a case of undiagnosed cutaneous leishmaniasis in a patient who received many weeks of inadequate antibiotic treatment. Ulceration in the right submandibular region was thought to be a purulent complication after laser surgery. Six weeks before the ulcer developed, the patient had visited the jungle (Guatemala). Cutaneous leishmaniasis was finally diagnosed after nine months based on a proper history and a polymerase chain reaction (PCR) assay. Treatment with antimony derivatives was administered. After three months, the ulcer healed but left a scar.

**Conclusion:**

A lack of knowledge about tropical diseases among doctors and an incomplete medical history were the reasons for many weeks of erroneous treatment of cutaneous leishmaniasis with antibiotics. This is the first reported case of cutaneous leishmaniasis misdiagnosed as a complication after an aesthetic medical procedure.

## Background

Cutaneous leishmaniasis is a tropical disease caused by a protozoan in the genus *Leishmania*. It is a transmissible disease and is carried by sand flies of the genera *Phlebotomus* (Mediterranean, Middle East) and *Lutzomyia* (Central and South America). The disease has been reported in 98 countries, and more than 350 million people are at risk of infection [1]. Approximately 2 million people worldwide are infected with leishmaniasis each year [2]. Leishmaniasis accounts for 5 to 10% of illnesses in tourists returning from the tropics [3].

Domestic (mainly dogs, rarely cats) and wild mammals, mainly foxes, are the reservoirs of leishmaniasis. *Leishmania* is an intracellular parasite of macrophages.

Cutaneous leishmaniasis is characterized by a long incubation period. A nonitchy papule appears after several weeks at the site of a bite from a *Leishmania*-infected sand fly, usually on an exposed part of the body. It then transforms into a nodule, from which asymptomatic ulceration develops, which enlarges without proper treatment. Usually, the adjacent lymph nodes are not enlarged.

There are three clinical forms of leishmaniasis: cutaneous, dermal-mucosal and visceral. Clinical forms are restricted to their area. There are several species of *Leishmania* that cause cutaneous leishmaniasis in humans, including *L. major*, *L. tropica*, *L. infantum*, *L. mexicana*, *L. amazonensis, L. braziliensis*, *L. guyanensis*, and *L. panamensis* [4].

## Case presentation

**Fig. 1.**
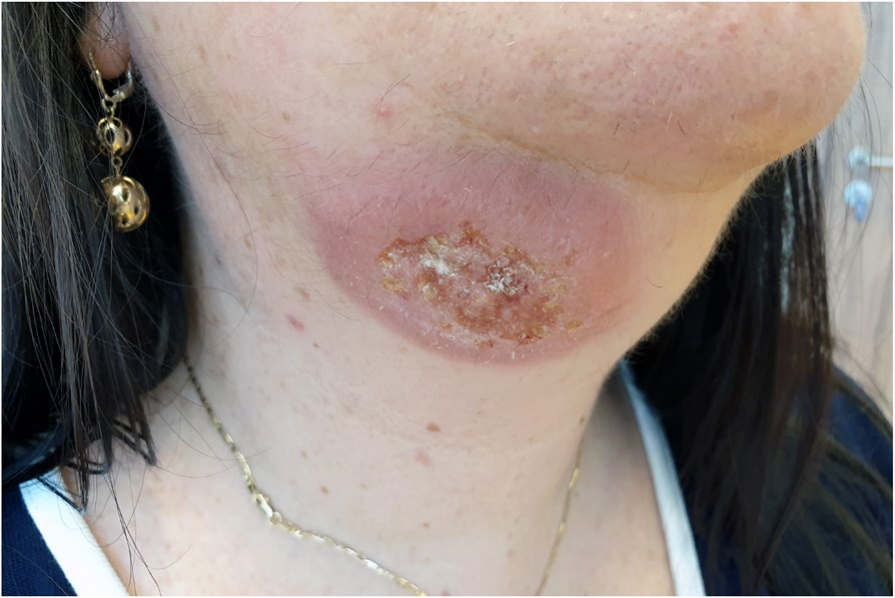
Non-painful ulceration covered by a 2 × 1 cm scab on erythematous in the right submandibular region five months after the onset of symptoms

A 52-year-old woman spent two weeks in Guatemala on a two-day jungle hike during which she was bit by insects. Five weeks after her visit to the jungle, she underwent advanced pulsed light (APL) laser hair removal in the neck area. One week after laser treatment, a nonitchy papule that was not associated with any other symptoms appeared in the right submandibular region; the nodule gradually enlarged and developed into a hard, dark red nodule with a soft edge. Over time, the nodule ulcerated and increased to 2 × 1 cm in size after five months (Fig. 1).

**Fig. 2.**
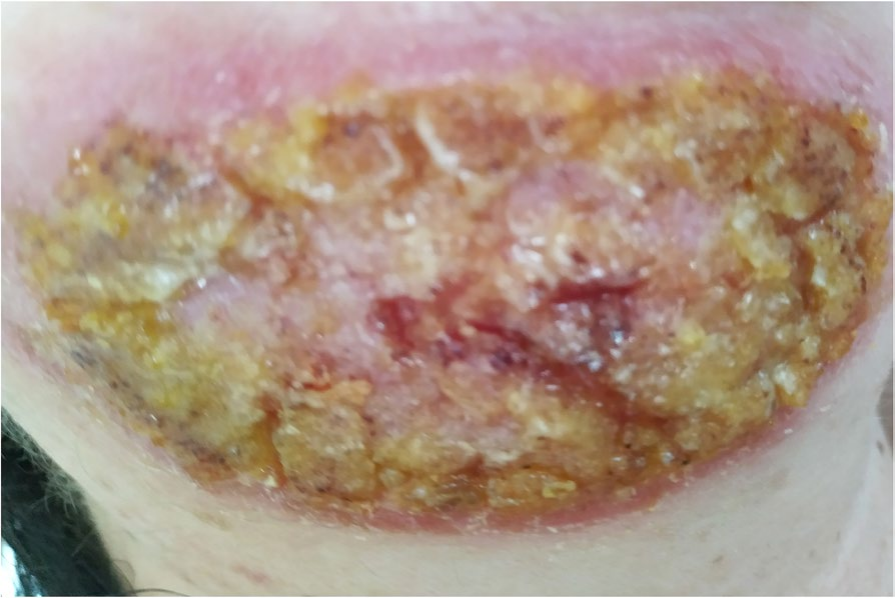
Eight months after the onset of symptoms, the ulceration had enlarged (10× 5 cm); it had an erythematous base and was covered with fibropurulent exudate and a thick scab on the periphery

Initially, bacterial infection was diagnosed by swabbing the ulcer and subjecting the swabs to fungal and bacterial culture, but the culture results were negative. Despite the negative result, the doctor recommended antibacterial treatment, including orally Augmentin 1.0 g (Amoxicillinum + Acidum Clavulanicum) for two weeks. Topical Mupirox 20 mg/g ointment was also applied. There was no improvement, and the patient visited a dermatologist and an ear, nose, and throat (ENT) specialist; these visits were followed by two hospital stays in the departments of infectious diseases and dermatology. A diagnosis of purulent dermatitis was made and topical and general antibiotics were continued (2% Argosulfan cream, 2% Detreomycin ointment), and general were continued (intravenously Taromentin were recommended for 2 weeks). The laboratory results were normal. Eight months after the onset of symptoms, the ulceration had enlarged (10× 5 cm); it had an erythematous base and was covered with fibropurulent exudate and a thick scab on the periphery (Fig. 2). The surrounding lymph nodes were not enlarged. The ulcer was swabbed for fungal and bacterial culture, which were both negative. Local and systemic antibiotic therapy consisting of 2% Detreomycin ointment and orally Ciprofloxacinum 0.5 g 2x/day was continued in the hospital setting for 7 days.

**Fig. 3.**
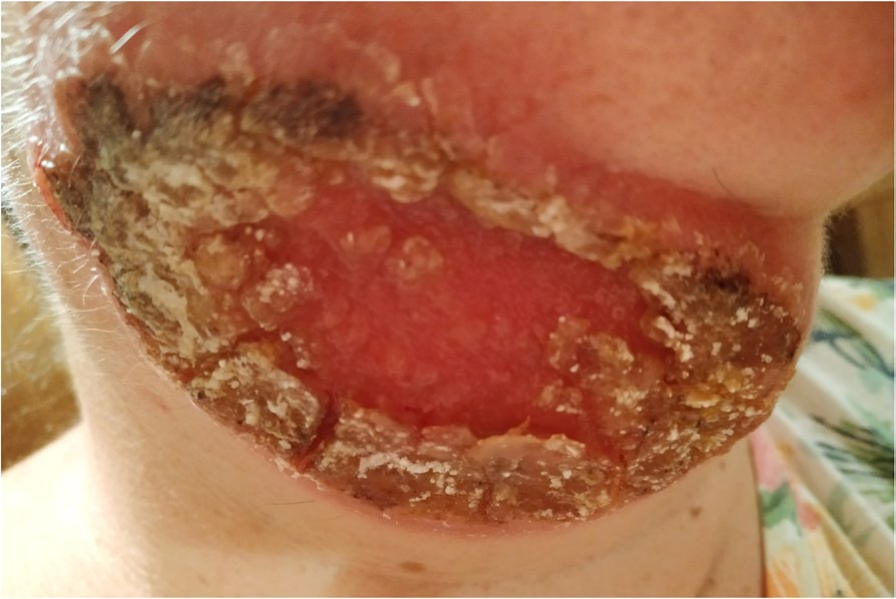
Nine months after symptom onset, the ulcer was large; it had an erythematous base and was covered with fibropurulent exudate and a thick scab on the periphery

Nine months after the onset of symptoms, the patient was hospitalized in the Department of Tropical and Parasitic Diseases at the University Centre for Maritime and Tropical Medicine in Gdynia. Nine months after symptom onset, the ulcer was large (10× 5 cm); it had an erythematous base and was covered with fibropurulent exudate and a thick scab on the periphery (Fig. 3).

**Fig. 4.**
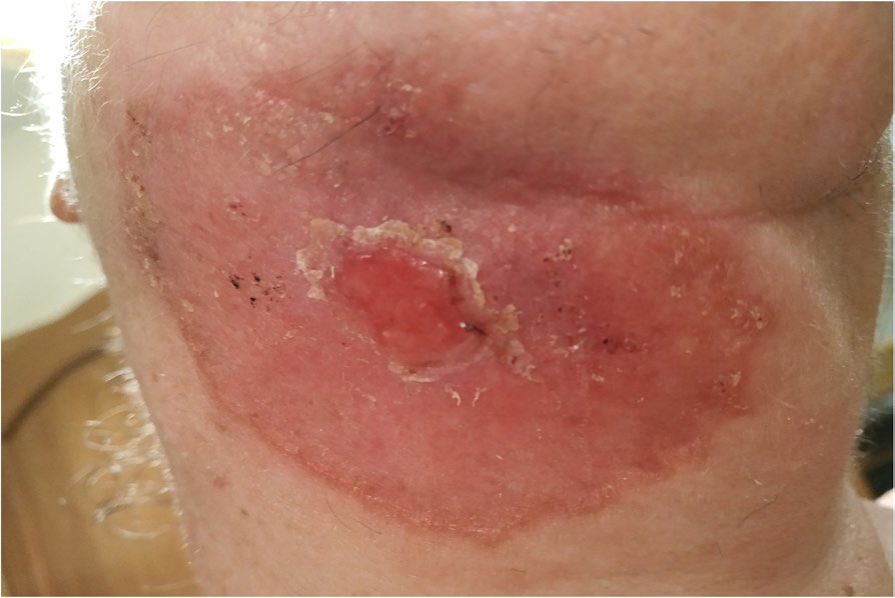
After cutaneous leishmaniasis was diagnosed the treatment were introduced; antimony derivatives (Glucantime 30 ampoules), local cryotherapy and 2% Argosulfan cream

**Fig. 5.**
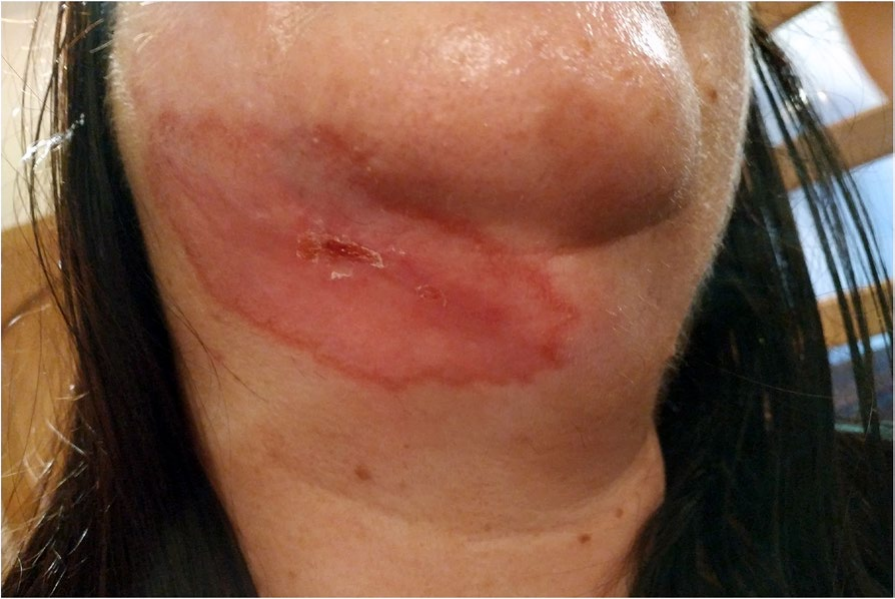
During treatment, there was visible gradual improvement

**Fig. 6.**
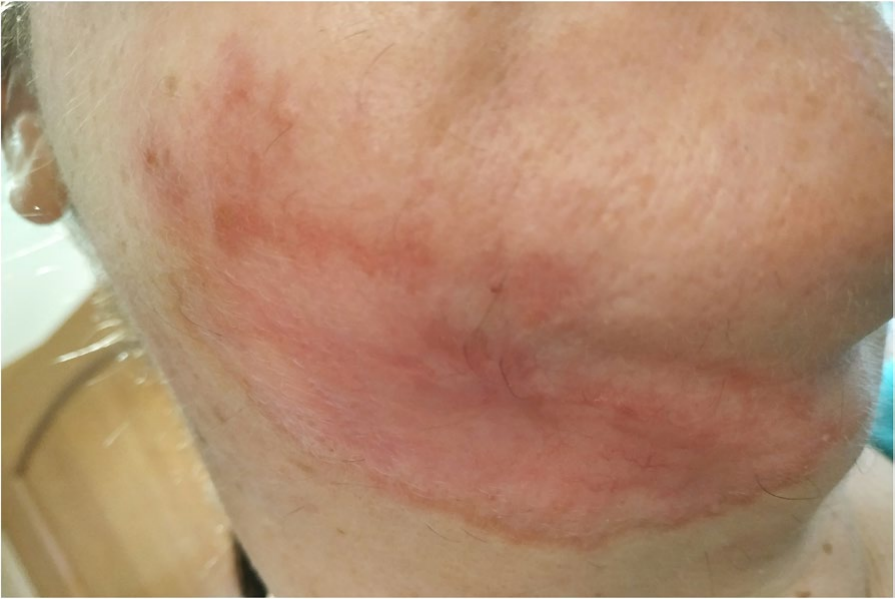
After treatment with antimony, the lesions healed but left an unsightly scar

On the basis of the patient’s travel history and the PCR findings, cutaneous leishmaniasis was diagnosed. Treatment with antimony derivatives intramuscularly (Glucantime 30 ampoules), local cryotherapy and 2% Argosulfan cream was administered. During treatment, there was gradual improvement (Figs. 4 and 5). After three months of treatment with antimony, the lesions healed but left an unsightly scar (Fig. 6).

## Discussion

The case presented herein is unique because the symptoms of cutaneous leishmaniasis were misdiagnosed as a complication after an aesthetic medicine procedure.

Failure to diagnose cutaneous leishmaniasis and an incomplete patient history led to many weeks of inadequate antibiotic treatment. Many researchers note the importance of collection of a thorough medical history in medical practice [5–8].

In the present case, the doctors initially diagnosed the ulcer as a complication of laser treatment, and for this reason, the patient was treated for several weeks with general and topical antibiotics. It was not until nine months later that she was referred to the Department of Tropical and Parasitic Diseases at the University Centre for Maritime and Tropical Medicine in Gdynia, where leishmaniasis was diagnosed by PCR with the following primers: LGITSF2 and LGITSR2 [9]. The composition of the reaction mixture was as follows: 12.5 μl of PCR Master MixPlus High GC (ready-to-use PCR mixture containing Taq DNA polymerase, PCR buffer, MgCl2 and dNTPs; A&A Biotechnology), 1 μl of each primer (concentration 10 μM) and 4 μl of DNA template, supplemented with deionized water up to 25 μl [10].

Proper diagnosis and treatment with antimony derivatives and cryotherapy resulted in a substantial improvement, and cure was achieved within three months. In the initial stage of cutaneous leishmaniasis, cryotherapy is very effective and safe [11, 12].

Approximately 1-10% of cases of cutaneous leishmaniasis caused by *L. braziliensis* in Southern and Central America may develop into the dermal-mucosal form [13–15]. The diagnosis of leishmaniasis is sometimes a difficult task, even for infectious disease physicians and dermatologists [16]. The problem of incorrect diagnosis and, consequently, incorrect treatment of cutaneous leishmaniasis has been described in Poland and other European countries [16–20].

Because of the various clinical manifestations, physicians in the Middle East and Central and South America, where leishmaniasis is endemic, also face challenges in making a correct diagnosis and thus administering appropriate treatment. Leishmaniasis cases have been misdiagnosed as skin cancer, lupus erythematosus, mycosis fungoides and tuberculosis [1, 15, 21]. As a result of travel and migration, leishmaniasis has been reported in countries that were previously nonendemic, e.g., Thailand [22].

## Conclusions

In this case, the patient first presented to the hospital four months after the appearance of a nonhealing ulceration in the right submandibular region. Laser treatment (APL) of the right submandibular region had no effect on the development of cutaneous leishmaniasis.

An incomplete history and the inexperience of the doctors in diagnosing tropical diseases were the reasons for inadequate treatment with antibiotics in this patient. Cutaneous leishmaniasis was finally diagnosed and treated with antimony derivatives after nine months based on a detailed medical history and PCR results.

This case is presented herein to disseminate knowledge about leishmaniasis and inform the medical community of the discussed issue. Specialist knowledge about tropical diseases and collection of a thorough medical history will contribute to the correct diagnosis and treatment of patients in medical practice [23].

### Strengths of the presented case

A case of a large ulcer in the submandibular region was presented. The presented case can increase awareness among the medical community regarding the diagnosis of leishmaniasis and the importance of collecting a thorough medical history.

### Limitations of the presented case

The patient was treated with long-term and ineffective antibiotics due to an incorrect diagnosis.

## Data Availability

Data supporting the findings are available upon request. Please contact the corresponding author, Katarzyna Van Damme-Ostapowicz (katarzyna.van.damme-ostapowicz@hvl.no), for data access.

## Abbreviations

APL: Advanced pulsed light
PCR: Polymerase chain reaction
